# Effectiveness of Low-Volume Versus High-Volume Ropivacaine for Ultrasound-Guided Maxillary Nerve Block in Double-Jaw Surgery: A Randomized Non-inferiority Trial

**DOI:** 10.1007/s00266-025-04671-9

**Published:** 2025-01-16

**Authors:** Ming-Kai Chen, Le Zhao, Wei Luo, Kai Luo, Jie Lin, Yang Ji

**Affiliations:** State Key Laboratory of Oral Diseases & National Center for Stomatology & National Clinical Research Center for Oral Diseases & Department of Anesthesiology, West China Hospital of Stomatology, Sichuan University, Chengdu, China

**Keywords:** Maxillary nerve block, Ropivacaine, Volume, Postoperative pain, Double-jaw surgery

## Abstract

**Background:**

Ultrasound-guided maxillary nerve block (UGMNB) is applied in oral and maxillofacial surgery to improve perioperative analgesia, decrease the risk of postoperative nausea and vomiting, and enhance recovery. However, the optimum volume of ropivacaine used for UGMNB is undetermined. Thus, it was hypothesized that in patients undergoing double-jaw surgery, low- and high-volume ropivacaine reduces perioperative pain with similar efficacy.

**Methods:**

Adults undergoing double-jaw surgery were enrolled in a randomized non-inferiority trial to receive a bilateral single-injection UGMNB with 2 mL (low-volume [LV] group) or 5 mL (high-volume [HV] group) of 0.375% ropivacaine on each side. A visual analog scale (VAS) score for maxillary pain at 2 h postoperatively was taken as the primary outcome. VAS score for maxillary and mandibular pain at 2, 4, 6, 8, 12, 24, and 48 h postoperatively, hemodynamic changes intraoperatively, consumption of intraoperative opioids and sedatives, vasoactive medication use, extubation time, postoperative rescue analgesia, time to the first analgesia, postoperative nausea and vomiting and UGMNB-related complications within 48 h post-surgery were assessed as the secondary outcomes.

**Results:**

Sixty-four adults were included. The maxillary pain score in the LV group was not inferior to that in the HV group at 2 h postoperatively, with a non-inferiority margin of 1 (mean difference − 0.1; 95% confidence interval [CI] − 0.6 to 0.8, *P *= 0.414 for non-inferiority). Maxillary and mandibular pain demonstrated no difference in the measured times between groups. The incidence of postoperative nausea was significantly higher in the LV group than that in the HV group at 6–24 h (12 (37.5%) vs. 5 (15.6%), *P *= 0.048). Moreover, no differences in intraoperative hemodynamic parameters, medications during anesthesia, time to extubation, rescue analgesia, time to the first analgesia, and postoperative vomiting were observed. Only one patient in the LV group was observed to have maxillary nerve block-related complications.

**Conclusions:**

To conclude, the efficacy of UGMNB with 2 mL of 0.375% ropivacaine has the same efficacy as the 5 mL drug in reducing perioperative pain in patients undergoing double-jaw surgery.

**Level of Evidence I:**

This journal requires that authors assign a level of evidence to each article. For a full description of these Evidence-Based Medicine ratings, please refer to the Table of Contents or the online Instructions to Authors www.springer.com/00266.

**Supplementary Information:**

The online version contains supplementary material available at 10.1007/s00266-025-04671-9.

## Introduction

In recent years, the management of perioperative pain for patients undergoing orthognathic surgery has become a significant concern [1]. Most patients undergoing double-jaw orthognathic surgery have reported moderate-to-severe acute pain postoperatively [2, 3]. Improper management of pain may be associated with reduced patient satisfaction [4–6], prolonged hospitalization [7], increased risk of adverse events (such as postoperative nausea and vomiting, [PONV]) [8], and development of chronic pain [9, 10]. Regional anesthesia is regarded as the core of multimodal analgesia according to the enhanced recovery after surgery (ERAS) concepts, modifying the current state of high-dose opioids based on the conventional double-jaw surgery regimes, and decreasing pain and opioid-related adverse events postoperatively [11].

Ultrasound-guided maxillary nerve block (UGMNB) is effectuated through the infra-zygomatic method. UGMNB can monitor local anesthetic spread within pterygopalatine fossa (PPF) and produce an exact effect of maxillary nerve block (MNB) based on the ultrasound images demonstrating the lateral pterygoid plate (LPP) and posterior maxillary edge as the landmarks [12]. Moreover, an uncertain effect of the Gasserian ganglion block or mandibular nerve block is also exhibited. This regional block technique is currently used for the diagnosis and treatment of trigeminal neuralgia [13] and perioperative analgesia in ear-nose-throat (ENT) [14] and maxillofacial surgeries [15].

Recently, Molins et al. [16] demonstrated that UGMNB with high-volume (5 mL) local anesthetic (LA) reduces the postoperative pain within 18 h following double-jaw surgery, while another study observed the decreased visual analog scale (VAS) pain score with the same volume of LA, within 12 h postoperatively [17]. A previous study demonstrated that UGMNB with low-volume (2 mL) LA provided an effective postoperative analgesic effect for 24 h [18]. A cadaveric anatomical study also suggested that the maxillary nerve can be effectively anesthetized using 1 mL of solution, while 5 mL results in negligent drug spread and non-specific nerve blockage [19]. These findings suggest that an HV (>2 mL) of LA may not be necessary under ultrasound guidance, and the effect of an MNB might not be altered markedly, inversely, with the increased risk of local anesthetics’ spread (e.g., pupil alteration, visual impairment, and non-specific nerve blockage). To date, effect of two commonly used volumes of a fixed concentration ropivacaine during UGMNB for patients undergoing double-jaw surgery has not been compared.

Therefore, we hypothesized that LV (2 mL) ropivacaine is non-inferior to HV (5 mL) drug in reducing perioperative pain.

## Methods

This randomized, parallel-controlled, double-blind, non-inferiority trial was conducted at West China Hospital of Stomatology, Sichuan University (Chengdu, Sichuan, China). The trial was approved by the Ethics Review Committee of West China Hospital of Stomatology (Approval number: WCHSIRB-D-2024-002-R2) and registered in the Chinese Clinical Trial Registry (Registration number: ChiCTR2400081389, registration time: 29/02/2024). Informed consent was obtained from all patients before enrollment.

### Patient Recruitment

Adults aged 18–45 years were screened to assess their eligibility for enrollment in this study with an American Society of Anesthesiologists classification status of I–II scheduled to undergo double-jaw surgery at West China Hospital of Stomatology (first enrollment: May 17, 2024). The exclusion criteria were as follows: patients with body mass index < 18 or > 30 kg/m^2^; those who required temporomandibular joint surgery; those with contraindications for controlled hypotension; those with established mental illness; those who were unable to communicate because of visual, hearing, speech, or other impairments; those with chronic pain or opioid abuse pre-surgery; those who were allergic to the drug components or ingredients used in this study; patients with contraindications for nerve block, including coagulation disorders, infection in the block site, and puncture route-related anatomical abnormalities; those with a history of severe bradycardia (heart rate [HR] ≤ 50 beats/min), those with different types of sinoatrial, intra-atrial, and intraventricular blocks, history of morbid sinus node syndrome or pre-excitation syndrome; and those who were planned to undergo submental endotracheal intubation rather than transnasal tracheal intubation.

### Randomization and Blinding

An online random digit generator (www.randomization.org) was used to generate random sequences with a block size of four at a ratio of 1:1. Following this, these random results were sealed in envelopes with sequential numbers and maintained by the trial coordinator. The coordinator then classified the participants into an LV (*n* = 32) or HV (*n* = 32) group. On the day of surgery, the envelopes were opened by the trial coordinator in the recruitment order, and the study drug was prepared according to the grouping scheme. In addition, each drug was similar in appearance and packaged in similar 20-mL syringes with the label “study medications.” A blinded anesthesiologist (WL) who was responsible for achieving the nerve block, received the dosing regimen. The trial coordinator did not participate in anesthetic procedures, perioperative care, or postoperative follow-up. The attending anesthesiologists (MC and LZ) in charge of anesthetic management, the surgical team, postoperative follow-up assessors (KL), and participants were blinded to randomized allocations until the statistical analysis was completed. Moreover, the primary researcher unmasked the blindness with respect to a patient in the LV group who was noted to have a severe MNB-related complication.

### Preoperative and Intraoperative Management

No presurgical medication was administered. The anesthetic protocol was standardized for all allocated patients in the study. Before anesthetic induction, preoxygenation was applied at 6 L/min by mask, and dexmedetomidine 0.5 ug/kg was infused for 10 min to blunt cardiovascular responses to endotracheal intubation. General anesthesia was induced by an intravenous injection of 1.5–2 mg/kg propofol, followed by 0.2–0.3 ug/kg sufentanil and completed with 0.2 mg/kg intravenous administration of cisatracurium to facilitate nasotracheal intubation. Then, 10 mg dexamethasone was routinely given to reduce postoperative tissue edema and PONV. After endotracheal intubation, mechanical ventilation was delivered at the 6–8 mL/kg tidal volume, 11–20 times/min respiratory rate, and the 1:2 inspiratory-to-expiratory time ratio to maintain the 35–40 mmHg end-tidal CO_2_. Before incision, to minimize bleeding, each surgical site was infiltrated using 10 mL of 1:400,000 epinephrine. When mandibular sagittal osteotomies were planned, the patients received the bilateral inferior alveolar nerve block by a surgeon using 5 mL of 1% lidocaine with 1:400,000 epinephrine. The anesthesia was maintained by sevoflurane titration to 1.5–2% end-tidal concentration for BIS values between 40 and 60, with continuous intravenous infusion of remifentanil (0.05–0.2 μg/[kg·min]) and dexmedetomidine (0.5 μg/[kg·h]). This was particularly performed in those who underwent maxillary osteotomies to eliminate surgical stress and reduce blood loss during surgery. Furthermore, the muscle relaxant cisatracurium was administered again during surgery, as needed. A controlled hypotension technique was applied during maxillary osteotomies to maintain mean arterial pressure (MAP) at 55–60 mmHg to reduce blood loss. The step-by-step procedure was as follows: (1) When the sevoflurane concentration and infusion speed of remifentanil reached the maximum for 10 min, 0.3 mg nicardipine was administered intravenously to achieve the targeted blood pressure and was repeated if required; (2) the first dose of 0.1 ug/kg sufentanil was administered when the blood pressure and HR began elevating consistently≥10%; (3) a bolus dose of 50 ug remifentanil was administered intravenously when blood pressure and HR persistently increased for > 5 min. The controlled hypotension method was applied until the completion of the maxillary osteotomy (lasting approximately 1 h), which was used neither during mandibular procedures nor during genioplasty. Following this, MAP was gradually increased to or > 80 mmHg. Ephedrine 3–6 mg or aramine 0.3 mg was administered if the MAP < 65 mmHg. Approximately 0.3–0.5 mg atropine was administered intravenously if the HR decreased to < 50 beats/min. Intraoperative crystalloid and colloid fluids were administered based on blood loss and urine output mediated by the attending anesthesiologist. Once the surgical suturing began, administration of both sevoflurane and dexmedetomidine was discontinued; however, that of propofol (4–6 mg/[kg·h]) continued throughout the surgical process. Moreover, remifentanil administration was discontinued 10 min before the end of surgery. The patients were extubated in the operating room, after regaining consciousness with spontaneous respiration. The patients were then transferred to the post-anesthesia care unit (PACU) for 2-h observation and later to the ward for continuous postoperative care.

### Surgery

The surgical procedure was standardized in our institution. Maxillary osteotomy was performed first in the surgery. A LeFort I osteotomy and a bilateral sagittal split osteotomy were performed on the maxilla and mandible, respectively.

### Ultrasound-Guided Maxillary Nerve Block

For both groups, participants underwent ultrasound-guided, bilateral, single injection of LAs in the PPF as previously described [20, 21]. The bilateral UGMNB was conducted by an experienced regional anesthesiologist (WL) following anesthesia induction in the operating room. Patients were positioned supine, with heads turned to the contralateral side. The portable ultrasound unit (Noblus, Hitachi, Chiba, Japan) with a 6.5 MHz convex array probe (C42, Fujifilm Healthcare Corporation, Chiba, Japan) was used to acquire ultrasound images. The probe was placed on one side of the patient’s face in the open-mouth posture, following aseptic skin preparation, followed by the transverse placement of the transducer beneath the zygomatic arch to recognize maxillary tuberosity and LPP. Following this, the probe was tilted from the caudal to the cranial direction until the top of the PPF was identified. The out-of-the-plane technique was adopted to guide a needle (22-G, 90 mm, AN-N, Zhengjiang, China) using real-time ultrasound to advance until the tip entered the PPF. To determine the needle tip correctly, 0.5 mL of 0.9% saline was injected, as necessary. A blinded solution (2 or 5 mL of 0.375% ropivacaine in both groups) was slowly injected on each side following the negative blood aspiration test.

### Postoperative Pain Management

All the participants received 40 mg parecoxib sodium and 0.05 ug/kg sufentanil for postoperative analgesia, approximately 30 min before the surgery completion. If the VAS was ≥ 4 cm and/or patients required analgesics during PACU, 0.05 ug/kg sufentanil was administered for rescue analgesics, if needed. Similarly, when the VAS was ≥ 4 cm and/or patients required analgesics in the ward, an additional 40 mg of parecoxib was administered intravenously, if necessary, every 6–12 h. However, when the VAS pain score was not mitigated and/or patients required analgesics in the ward (≥ 4 cm at 30 min following rescue analgesics), administration of 50 mg of tramadol was planned.

### Outcome Measurements

All participants were followed up for 48 h after surgery. The risk of postoperative nausea and/or vomiting was calculated based on the Apfel score, and the risk factor of a score of 0-1, 2, or 3-plus can be divided into “low-,” “medium-,” or “high-” risk categories, respectively [22]. The pain was evaluated using a 10-cm VAS, which was categorized as no pain (0), mild (1–3), moderate (4–6), and severe (7–10) [23]. PONV was defined as the early (1–6 h) or late phase (6–24 h) after a surgical procedure [24]. The VAS score for maxillary pain at 2 h postoperatively was the primary study outcome. The secondary outcomes were as follows: (1) VAS score at 2, 4, 6, 8, 12, 24, and 48 h postoperatively for maxillary and mandibular pain; (2) intraoperative hemodynamic changes (HR, MAP, SBP, and DBP) at 6-time points, namely T0, baseline; T1, 1 min after intubation; T2; after incision; T3, 1 min before maxillary osteotomy; T4, the maximum value measured during maxillary down-fracture; T5, 1 min before mandibular osteotomy; and T6, the maximum value measured during mandibular osteotomy; (3) intraoperative opioids and sedative consumption; (4) the use of vasoactive medications; (5) the time to extubation; (6) rescue analgesics within 48 h postoperatively; (7) time to the first analgesia; (8) incidence of PONV; and (9) MNB-related complications within 48 h post-surgery such as dizziness, headache, visual impairment, hematoma, pupil alteration and so on.

### Statistical Analysis

Statistical analysis was conducted using SPSS 26.0 (IBM, Chicago, IL, USA). Data of normal distribution were indicated by mean ± standard deviation (SD), while those of non-normal distribution were represented by median (lower quartile, upper quartile), and categorical data were indicated by frequencies (%). Continuous variables were compared between the groups using Student’s *t* test or the Mann–Whitney U test, based on the normality of distribution as determined by the Kolmogorov–Smirnov test, whereas categorical variables were analyzed using the *χ*^2^ test or Fisher’s exact test. For ordinal data, differences were assessed using the Mann–Whitney *U* test. For the primary outcome, the pain scores between low- and high-volume groups were compared using the Mann–Whitney *U* test. The non-inferiority hypothesis for the primary outcomes was examined using a one-sided *t* test or Mann–Whitney *U* test at a significance level of *α* =0.025. For the predetermined non-inferiority margin and null hypothesis, we presented the two-sided 95% CI, the upper margin of which was equivalent to the upper margin of the one-sided 97.5% CI of the difference in pain scores by treatment. A nonparametric Mann–Whitney *U* test was conducted to test the hypothesis that the pain difference at each time point between the two groups is significant. The difference in rating pain score within each treatment group postoperatively at different time points was assessed by the nonparametric Friedman test. Comparisons of pain scores for maxillary and mandibular pain over seven-time points were also made using a generalized estimating equation (GEE) model. The changes in vital signs (HR, MAP, SBP and DBP) over time between the groups were compared using a two-way repeated-measures analysis of variance (ANOVA) with group (two levels: low- and high volume) and time (seven levels: T0, T1, T2, T3, T4, T5 and T6) as within-subject factors. Bonferroni correction was performed in multiple comparisons. The minimal clinically important difference for postoperative pain is defined as 1 [25]. Statistical significance was set at *P*< 0.05.

### Sample Size Calculation

The sample size was determined using PASS 26 software (NCSS, Kaysville, Utah, USA). Based on the literature review [25], the strict non-inferiority margin is selected as 1, which is set in the analysis for differences in the means of two groups (low- minus high-volume group); this clinically relevant difference was referred to as the VAS score for maxillary pain between groups at 2 h postoperatively. Based on the institutional electronic health record software and our recently completed data, the standard deviation of 2-h VAS postoperatively was 1.3. To achieve 80% power while demonstrating non-inferiority, a sample size of 64 cases (32 in each group) was required to provide 0.8 power and 0.025 one-sided *α* value with a 10% dropout possibility.

## Results

**Fig. 1:**
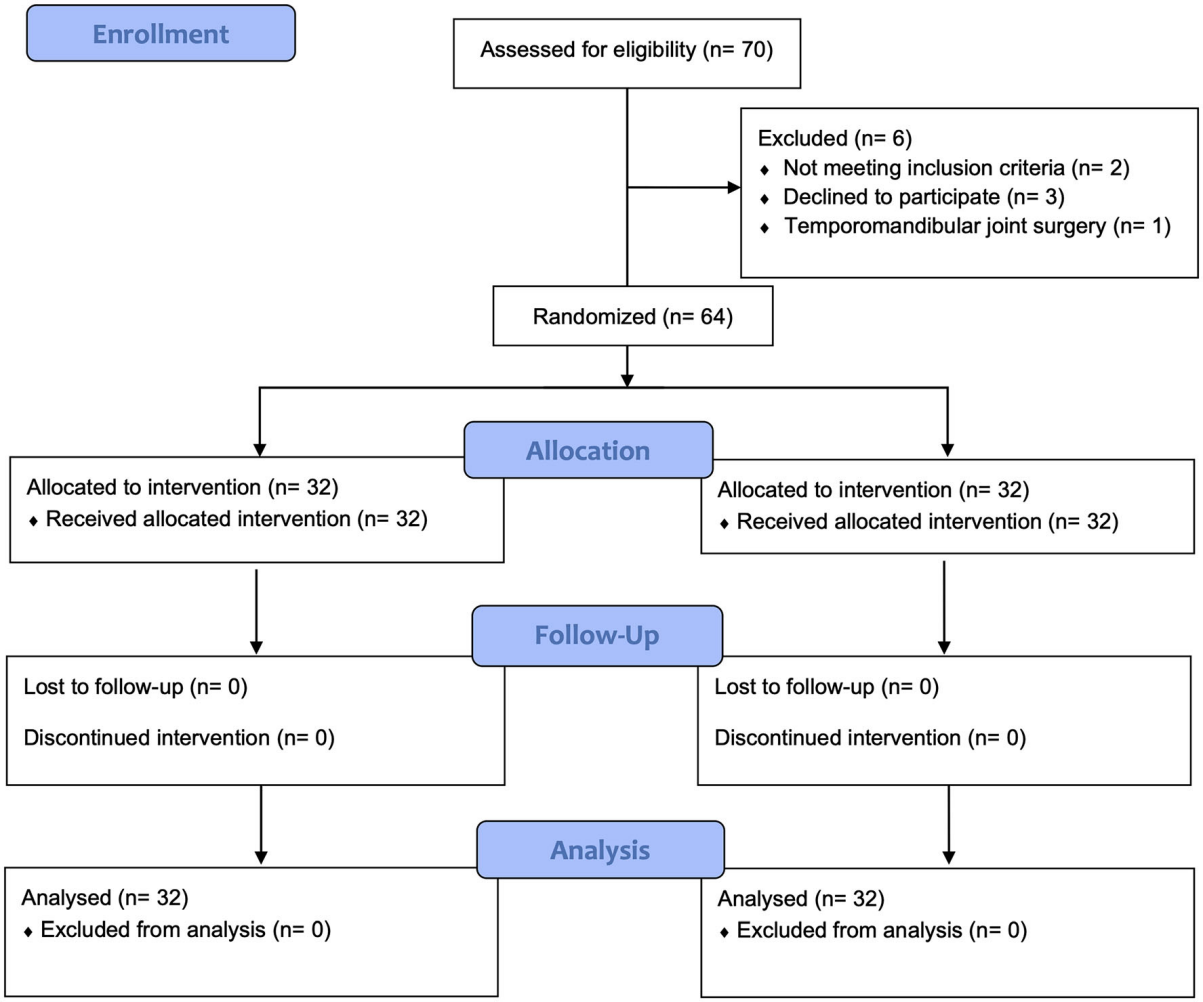
CONSORT flow diagram

From May 2024 to September 2024, a total of 70 patients aged 18 to 45 years scheduled for double-jaw surgery at our institution were recruited. After applying the exclusion criteria, 64 patients were finally included in this study, with 32 patients in each group. No patient was excluded or lost to follow-up. Figure 1 illustrates the flow chart of the study. Patient characteristics were comparable between the two groups (Table 1).

**Table 1:** Baseline characteristics of study participants

Variable	Low volume (*n *= 32)	High volume (*n *= 32)	*P* value
Age, years	23.50 (21.00–28.00)	22.00 (20.00–27.00)	0.134
Sex, *n* (%)			0.606
Female	21 (65.6)	19 (59.4)	
Male	11 (34.4)	13 (40.6)	
Height, cm	168.78 ± 8.81	166.72 ± 8.38	0.341
Weight, kg	58.19 ± 10.65	58.28 ± 9.65	0.971
BMI, kg/m^2^	20.30 ± 2.61	20.97 ± 3.23	0.367
ASA status			0.172
I	25 (78.1)	29 (90.6)	
II	7 (21.9)	3 (9.4)	
Apfel score, *n* (%)			0.636
0	4 (12.5)	1 (3.1)	
1	7 (21.9)	8 (25.0)	
2	10 (31.3)	12 (37.5)	
3	11 (34.4)	12 (34.4)	

Values are mean ± SD, number (proportion) or median (IQR). *BMI* body mass index; *ASA* American Society of Anesthesiologists

**Fig. 2:**
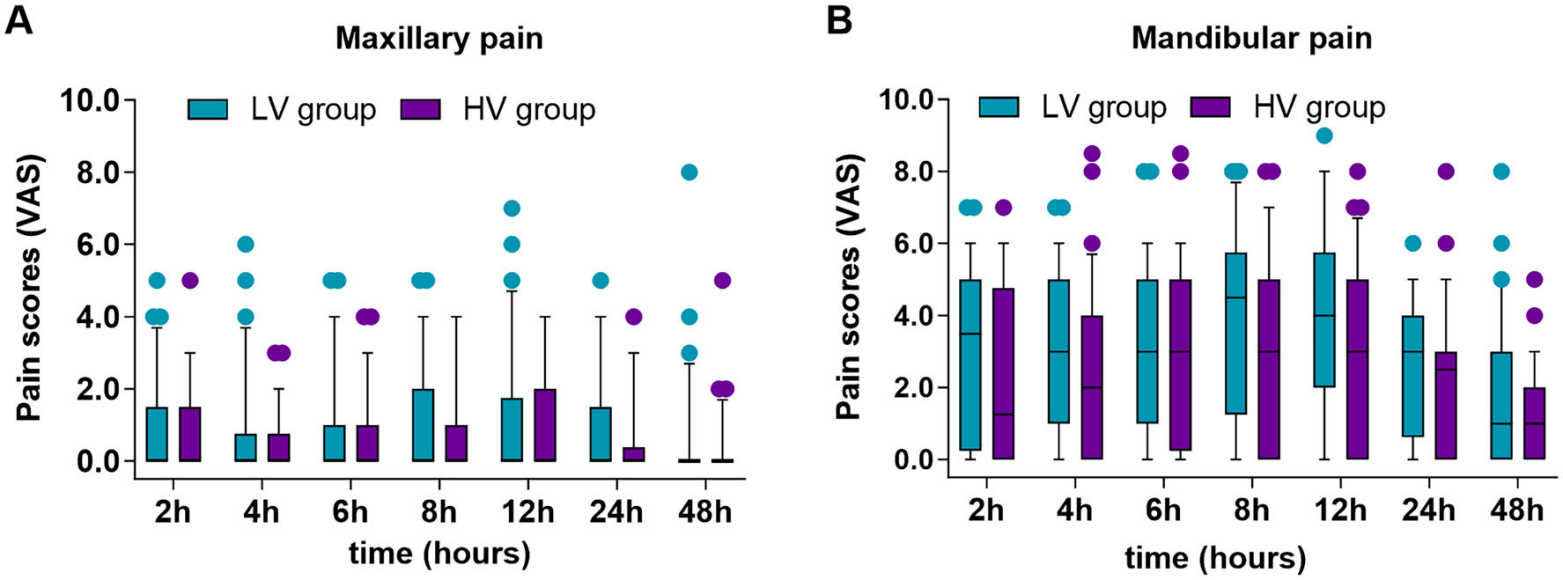
Comparison of VAS scores for **A** maxillary pain and **B** mandibular pain between the two groups. This measurement was repeated at 2, 4, 6, 8, 12, 24, 48 h postoperatively. Scores are presented as median (IQR), 25–75th percentiles (boxes), 10–90th percentiles (bars), and extremes (filled circles). LV, low volume; HV, high volume; VAS, visual analog scale

**Fig. 3:**
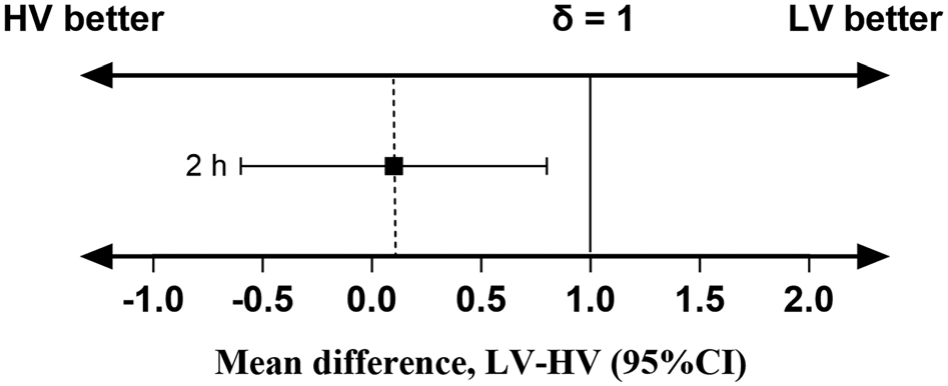
Mean differences for VAS pain scores with the non-inferiority margin *δ *= 1. Error bars represent 95%CIs. *LV* low volume; *HV* high volume

UGMNB was successfully performed on all study participants. Intra- and postoperative outcomes are presented in Table 2. The median (interquartile range) VAS score for maxillary pain at 2 h after surgery when comparing patients in the LV group vs. HV group was 0 (0–1.5) vs. 0 (0–1.5) (Fig. 2A), with a mean (SD) of 0.8 (1.5) vs. 0.7 (1.3). Moreover, the LV group did not exhibit inferiority to the HV group (0.1 [95% CI −0.6 to 0.8]), while the 95% CI for the mean treatment difference (LV minus HV) did not cross the non-inferiority margin of *δ*= 1 (*P*= 0.414) (Fig. 3). Both groups had relatively low scores for postoperative pain outcomes (Table S3). Repeated measurements demonstrated that the main effects of group (*P*= 0.470), time (*P *= 0.286), and group*time interactions (*P *= 0.736) between the two groups were not significantly different regarding the VAS scores in the maxilla (Table S1). The GEE revealed no significant group effects (*P* = 0.161) and group-time interaction (*P* = 0.463) between both groups when considering the VAS scores in the mandible in the measured times; only a significant main effect of time within each group was observed (*P *< 0.001) (Table S2). The peak period for the onset of mandibular pain was within the first 8 h after surgery, continuing until 24 h, and up to 48 h after surgery. Pain scores were approximately 2–3 points higher in the mandible than those in the maxilla (Table S3). The pain intensity difference (mandibular pain scores minus maxillary pain scores) was not statistically significant between the two groups (Table S4).

**Table 2:** Comparison of intra- and postoperative outcomes between the two groups

Outcomes	Low volume (*n *= 32)	High volume (*n *= 32)	*P* value
Intraoperative drugs			
Dose of remifentanil, ug/kg/min	0.07 ± 0.02	0.07 ± 0.02	0.760
Dose of sufentanil, ug	21.50 (17.50, 23.38)	18.16 (17.50, 22.50)	0.121
Dose of propofol, mg	425.96 ± 109.91	420.24 ± 137.88	0.855
Dose of dexmedetomidine, ug/kg/h	0.36 ± 0.06	0.37 ± 0.06	0.290
Dose of sevoflurane, ml	40 (40, 50)	40 (40, 50)	0.642
Use of vasopressor, *n* (%)	20 (62.5)	19 (59.4)	0.798
Use of depressor, *n* (%)	11 (34.4)	10 (31.3)	0.790
Duration of surgery, min	265.0 (253.0, 288.8)	252.0 (230.8, 296.5)	0.277
Duration of anesthesia, min	309.0 (283.5, 337.5)	300.00 (277.5, 346.5)	0.752
Time to extubation, min	8.8(5.0, 12.0)	6.5 (3.3, 10.8)	0.208
Rescue analgesic, *n* (%)	13 (40.6)	9 (28.1)	0.292
Time to first analgesia (min)	470 ± 256	627 ± 255	0.130
PON			
0–6 h	16 (50.0)	9 (28.1)	0.073
6–24 h	12 (37.5)	5 (15.6)	**0.048**
24–48 h	0 (0)	0 (0)	N/A
POV			
0–6 h	11(34.4)	7(21.9)	0.266
6–24 h	9(28.1)	5(15.6)	0.226
24–48 h	0 (0)	1 (3.1)	1.000

Bold value indicates significant differences (*P* < 0.05)

Values are presented as values are mean ± SD, number (proportion), or median (IQR)

**Fig. 4:**
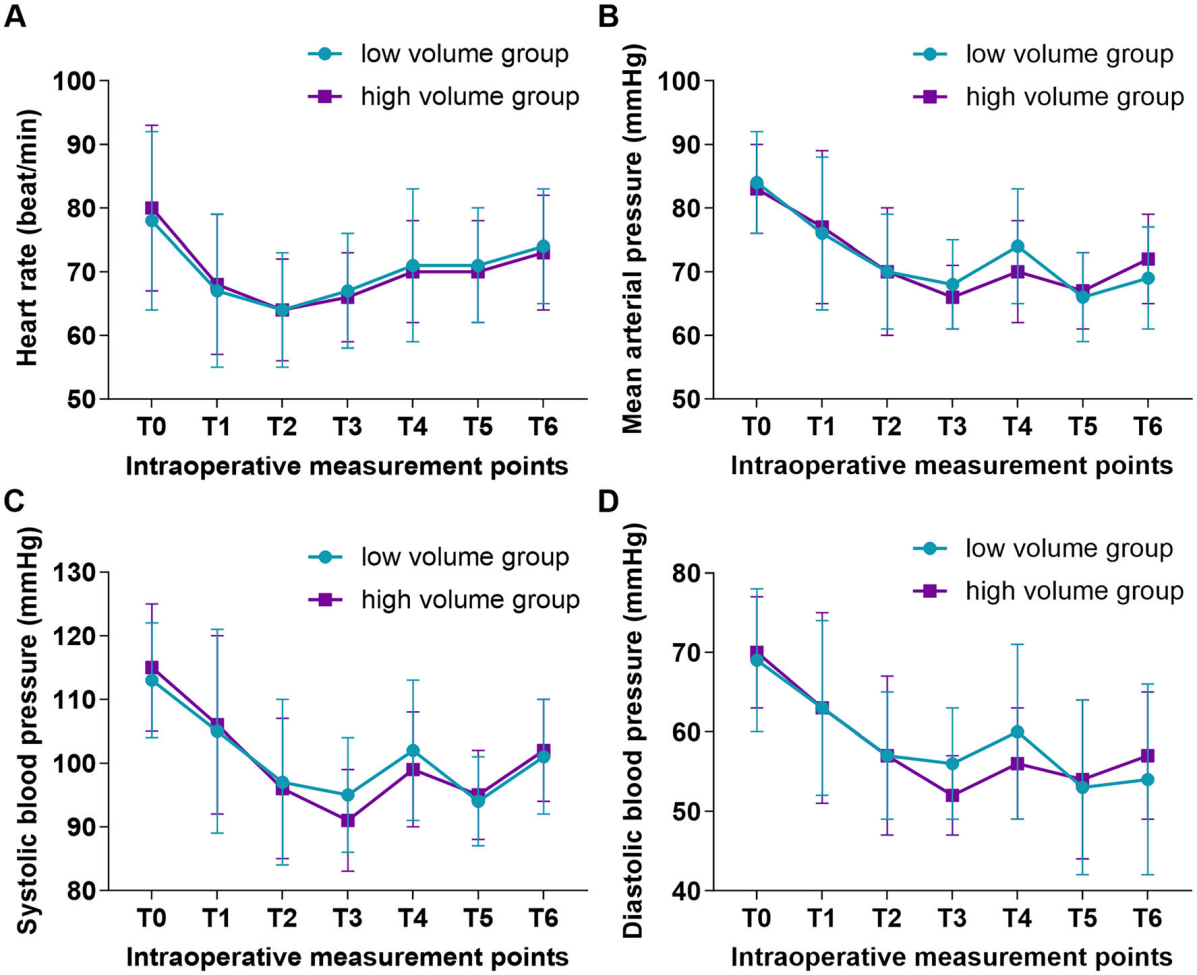
Intraoperative hemodynamic changes at six-time points: **A** HR; **B** MAP; **C** SBP; **D** DBP. T0, baseline; T1, 1 min after intubation; T2; after incision; T3, 1 min before osteotomy of the maxilla; T4, the maximum value measured during maxillary down-fracture; T5, 1min before osteotomy of the mandible; T6, the maximum value measured during osteotomy of the mandible

No significant group effects between both groups were observed for the intraoperative hemodynamic parameters (HR, MAP, SBP, and DBP) (HR, *P *= 0.950; MAP, *P *= 0.977; SBP, *P *= 0.946; DBP, *P *= 0.713), only a significant time main effect of time within each group was noted (HR, *P *< 0.001; MAP, *P *< 0.001; SBP, *P *< 0.001; D*BP*, *P *< 0.001) (Tables S5–S8; Fig. 4A–D). There was no significant difference between intraoperative data, including medications during anesthesia, durations of anesthesia and surgery, time to extubation, rescue analgesic, and time to the first analgesia among the two groups (Table 2). The incidence of nausea at 6–24 h post-surgery was significantly higher in the LV group than that in the HV group (*P *= 0.048; Table 2). However, the administration of analgesics did not demonstrate any significant correlation with the increased risk of postoperative nausea (PON) (Pearson’s correlation coefficient of 0.242, *P *= 0.623). No significant difference between the two groups was observed for postoperative vomiting at 0–6, 6–24, and 24–48 h (Table 2).

A total of 12 puncture-site bleeding was observed (five and seven in the LV and HV groups, respectively). Three local hematomas (two in the LV group and one in the HV group), located in the masseter muscle, were also observed. All episodes ceased after hard external compression for a few minutes. Postoperative complete awakening from anesthesia, a 36-year-old female patient in the LV group was noted with left-sided complete ptosis and numbness in the distribution of the ophthalmic branch of the trigeminal nerve. The ocular examination revealed an equally round pupil, reactive to light, with normal visual acuity and full extraocular movements in all directions. No evidence of facial nerve palsy, myasthenia gravis, or Horner’s syndrome was noted. However, evidence to support the aforementioned clinical manifestations in the craniocerebral computed tomographic examination postoperatively was unavailable. The patient was assured by the medical staff. These symptoms had spontaneously and completely disappeared approximately 24 h post-surgery; however, a new onset of pain in the maxilla and mandible was 4 and 5 points, respectively.

## Discussion

This study suggested that LV ropivacaine is non-inferior to HV drugs for 2-h VAS pain scores in the maxilla. Both groups could achieve prolonged analgesia in the maxilla (> 24 h). However, no difference between the groups was observed for hemodynamic changes, consumption of intraoperative opioids and sedatives, and vasoactive drug use intraoperatively. Postoperatively, no significant difference was observed in maxillary and mandibular pain at different time points, rescue analgesics, time to the first analgesia, and vomiting; however, a higher incidence of nausea (6–24 h) was observed in the LV group.

Both groups demonstrated similar pain intensity and trajectory. These results suggested that the low-volume local anesthetic is sufficient to encompass and block the maxillary nerve. Moreover, patients experienced mild to moderate pain postoperatively, which was consistent with the findings reported in previous studies [16–18]. An MNB can be performed by injecting an LA into PPF to produce an exact effect of the blockage of maxillary nerve. The average PPF volume is reported as 0.7–1.2 mL among adults, which may be unaffected by cephalic and upper facial indexes [19]. A previous cadaveric anatomical study recommended that 1 mL of solution was sufficient to encompass the maxillary nerve, whereas 5 mL resulted in negligent drug spread as well as non-specific nerve blockage [19]. For example, 5 mL of the LA not only fills the PPF and spreads in the infratemporal fossa or above the temporalis muscle. This results in blockage of the mandibular nerve branches, deep temporal nerves, and masseteric nerves [19]. Therefore, a 2–5 mL injection volume is substantially beyond the PPF cavity. One patient in the LV group in this study was also noted with prolonged left-sided ptosis and numbness in the distribution of the ophthalmic nerve. These ocular complications (e.g., pupil alteration, diplopia, and ptosis) may be elucidated by injected LA volume impact that may spread to the orbit or intracranial space [26]. Overall, UGMNB is a safe and efficacious strategy for pain management, and most ocular complications are transient and benign [26, 27]. A lower injectate volume (1.5 mL) of LA can also lead to MNB-related ocular complications [28]. Another retrospective study among 411 participants found no significant adverse effects from UGMNB with a high volume of LA (5 mL) [27]. However, no ocular complications in the HV group were found in this study. This may be elucidated by the small sample size and the fact that the complications were not accurately observed under general anesthesia. Based on the aforementioned results, a high volume (> 2 mL) of LA may not be necessary under ultrasound guidance. In addition, the effect of MNB might not be altered markedly and inversely, with the increased risk of spread of LA (e.g., pupil alteration, visual impairment, and non-specific nerve blockage).

The pain scores were approximately 2–3 points higher in the mandible than those in the maxilla, and the difference in pain intensity in the maxilla and mandible (mandibular pain scores minus maxillary pain scores) between groups was similar. This might be owing to the MNB reasonably expected to provide a better analgesic effect in the maxilla than that in the mandible. Thus, larger volumes of LAs (> 5 mL) are required to achieve the blockage of the mandibular nerve and its branches from an anatomical point of view [19]. To the best of our knowledge, this is the first study to report the use of LV and HV of LAs and validate them in double-jaw surgery.

We observed that intraoperative hemodynamic changes, especially during maxillary (T3–T4) and mandibular osteotomy (T5–T6), and opioid consumption were similar between the groups. This might suggest equivalent intraoperative analgesic efficacy between LV and HV LA for UGMNB. A blank control group to prove the effect of UGMNB was lacking. Previous studies have demonstrated the analgesic effects of both LV and HV drugs for UGMNB during surgery [16–18].

The incidence of PON in 6–24 h in this study is 37.5% compared to the HV group (15.6%). However, this discrepancy cannot be elucidated completely. Nausea is a self-reported subjective feeling that is affected by postoperative variable factors, such as the block technique, postoperative pain management (e.g., use of parecoxib), and patients’ self-oral care. Furthermore, these data should be interpreted with caution because of the possibility of small-study effects or recall bias. Although parecoxib was administered for rescue analgesia at our institution, it can also cause nausea because of injuries to the gastric mucosa [29]. However, no correlation was observed between the use of analgesics and PON in 6–24 h. All the participants were shifted to the ward 2 h after surgery. Thus, we speculate additional potential confounding factors that might have contributed to this difference, such as swallowing blood [30], and nausea induced by drinking [31].

This study has some limitations. First, the pain intensity is nonlinear, and subtle changes cannot be expressed by patients under distress because the pain score is not sensitive. Second, this study lacked a blank control group; therefore, the extent to which the two different volumes of ropivacaine for MNB would produce analgesia in the maxilla and mandible was not determined. This may have limited the depth of our study. Third, this study only assesses the pain in the early postoperative period. However, a long-term follow-up to monitor the effect of MNB with ropivacaine on chronic postoperative pain was not performed. Finally, this study might have underestimated the true incidence of nerve block-related complications because symptoms of those could hardly be observed under general anesthesia.

## Conclusions

In conclusion, this study suggests that an LV (2 mL) ropivacaine for UGMNB in double-jaw surgery could provide intra- and postoperative analgesia similar to HV (5 mL) injections. The LV group showed no increase in intraoperative opioid consumption and exhibited stable hemodynamic fluctuations, similar to those observed in the HV group. Thus, UGMNB with an LV (2 mL) of LA may be necessary to reduce the risk of LAs’ spread (e.g., pupil alteration, visual impairment, and non-specific nerve blockage). Future evidence is required to establish the safety and efficacy of LA, aiming to identify the optimal concentration and dose for UGMNB in oral and maxillofacial surgery.

## Supplementary Information

Below is the link to the electronic supplementary material.

Supplementary file1 (DOCX 27 KB)
