# Supplementary material for: Effectiveness of Low-Volume Versus High-Volume Ropivacaine for Ultrasound-Guided Maxillary Nerve Block in Double-Jaw Surgery: A Randomized Non-inferiority Trial
Source: Aesthetic Plast Surg. 2025 Jan 16;49(5):1217–26. doi: 10.1007/s00266-025-04671-9 (PMC11965159; doi:10.1007/s00266-025-04671-9)
Supplement: Supplementary file 1 — Supplementary file1 (DOCX 27 KB) [file 266_2025_4671_MOESM1_ESM.docx]

**Table S1** The VAS score for maxillary pain

| Group | 2 h | 4 h | 6 h | 8 h | 12 h | 24 h | 48 h | *χ^2^* | *P* value |
| --- | --- | --- | --- | --- | --- | --- | --- | --- | --- |
| low volume group | 0.00 (0.00, 1.50) | 0.00 (0.00, 0.75) | 0.00 (0.00, 1.00) | 0.00 (0.00, 2.00) | 0.00 (0.00, 1.75) | 0.00 (0.00, 1.50) | 0.00 (0.00, 0.00) | 2.330 | 0.887 |
| high volume group | 0.00 (0.00, 1.50) | 0.00 (0.00, 0.75) | 0.00 (0.00, 1.00) | 0.00 (0.00, 1.00) | 0.00 (0.00, 2.00) | 0.00 (0.00, 0.375) | 0.00 (0.00, 0.00) | 12.851 | 0.045 |
| Mean difference (95%CI) | 0.1 (-0.6, 0.8) | 0.4 (-0.3,1.1) | 0.2 (-0.5, 0.9) | 0.2 (-0.6, 0.9) | 0.1 (-0.8, 1) | 0.3 (-0.4, 0.9) | 0.3 (-0.4, 0.9) |  |  |
| *P* value | 0.923 | 0.704 | 0.852 | 0.857 | 0.750 | 0.798 | 0.875 |  |  |
| Group | *χ^2^*= 0.521, *P*= 0.470 | | | | | | | | |
| Time | *χ^2^*= 7.393, *P*= 0.286 | | | | | | | | |
| Interaction | *χ^2^*= 3.557, *P*= 0.736 | | | | | | | | |

Values are presented as median (IQR). The changes in VAS score for maxillary pain over time were analyzed using the Friedman test for all timepoints. The changes in VAS score for maxillary pain over time between the two groups were compared using generalized estimating equation model. The pain difference at each time points between the two groups were compared using Mann-Whitney U-test.

**Table S2** The VAS score for mandibular pain

| Group | 2 h | 4 h | 6 h | 8 h | 12 h | 24 h | 48 h | *χ^2^* | *P* value |
| --- | --- | --- | --- | --- | --- | --- | --- | --- | --- |
| low volume group | 3.5 (0.3, 5.0) | 3.0 (1.0, 5.0) | 3.0 (1.0, 5.0) | 4.5 (1.3, 5.8) | 4.0 (2.0, 5.8) | 3.0 (0.6, 4.0) | 1.0 (0.0, 3.0) | 53.438 | <0.001 |
| high volume group | 1.3 (0.0, 4.8) | 2.0 (0.0, 4.0) | 3.0 (0.3, 5.0) | 3.0 (0.0, 5.0) | 3.0 (0.0, 5.0) | 2.5 (0.0, 3.0) | 1.0 (0.0, 2.0) | 38.756 | <0.001 |
| Mean difference (95%CI) | 0.9 (-0.2, 2.1) | 0.6 (-0.5, 1.8) | 0.2 (-1, 1.4) | 0.9 (-0.4, 2.2) | 1.1 (-0.2, 2.3) | 0.3 (-0.7, 1.2) | 0.5 (-0.4, 1.4) |  |  |
| *P* value | 0.120 | 0.198 | 0.659 | 0.127 | 0.094 | 0.387 | 0.488 |  |  |
| Group | *χ^2^*= 1.964, *P*= 0.161 | | | | | | | | |
| Time | *χ^2^*= 70.935, *P*< 0.001 | | | | | | | | |
| Interaction | *χ^2^*= 5.655, *P*= 0.463 | | | | | | | | |

Values are presented as median (IQR). The changes in VAS score for mandibular pain over time were analyzed using the Friedman test for all timepoints. The changes in VAS score for mandibular pain over time between the two groups were compared using generalized estimating equation model. The pain difference at each time points between the two groups were compared using Mann-Whitney U-test.

**Table S3** The difference in VAS scores between maxillary pain and mandibular pain over time

| Location | 2 h | 4 h | 6 h | 8 h | 12 h | 24 h | 48 h |
| --- | --- | --- | --- | --- | --- | --- | --- |
| Maxillary pain | 0.8 ± 1.4 | 0.7 ± 1.4 | 0.8 ± 1.5 | 0.9 ± 1.6 | 1 ± 1.7 | 0.7 ± 1.3 | 0.5 ± 1.4 |
| Mandible pain | 2.6 ± 2.4 | 2.7 ± 2.3 | 3.2 ± 2.4 | 3.5 ± 2.6 | 3.4 ± 2.6 | 2.5 ± 1.9 | 1.5 ± 1.8 |
| Mean difference (95%CI) | -1.9 (-2.5, -1.2) | -2 (-2.7, -1.4) | -2.4 (-3, -1.8) | -2.6 (-3.3, -1.9) | -2.5 (-3.2, -1.7) | -1.8 (-2.4, -1.2) | -1 (-1.5, -0.4) |
| *P* value | <0.001 | <0.001 | <0.001 | <0.001 | 0.001 | 0.002 | 0.001 |

Values are presented as mean ± SD. The VAS scores at specific time point between groups were compared using independent t-test.

**Table S4** The VAS score difference between the two groups over time

| Group | 2 h | 4 h | 6 h | 8 h | 12 h | 24 h | 48 h |
| --- | --- | --- | --- | --- | --- | --- | --- |
| low volume group | 2.3 ± 2.2 | 2.2 ± 2 | 2.4 ± 2 | 3 ± 2.5 | 2.9 ± 2.3 | 1.8 ± 1.9 | 1.1 ± 2 |
| high volume group | 1.4 ± 2.8 | 1.9 ± 2.5 | 2.4 ± 2.6 | 2.2 ± 2.8 | 2 ± 2.8 | 1.8 ± 2.3 | 0.9 ± 1.7 |
| *P* value | 0.179 | 0.681 | 0.936 | 0.266 | 0.132 | 0.977 | 0.642 |
| Group | *F*= 0.884, *P*= 0.351 | | | | | | |
| Time | *F*= 8.096, *P*< 0.001 | | | | | | |
| Interaction | *F*= 1.112, *P*= 0.351 | | | | | | |

Values are presented as mean ± SD. The mean difference scores (mandibular pain minus maxillary pain) at specific time point between groups were compared using independent t-test. A two-way repeated measures analyses of variance were also performed with group and time as factors.

**Table S5** The changes in heart rate in both groups at 7 measurement points

| Group | T0 | T1 | T2 | T3 | T4 | T5 | T6 |
| --- | --- | --- | --- | --- | --- | --- | --- |
| low volume group (n=32) | 78 ± 14 | 67 ± 12 | 64 ± 9 | 67 ± 9 | 71 ± 12 | 71 ± 9 | 74 ± 9 |
| high volume group (n=32) | 80 ± 13 | 68 ± 11 | 65 ± 8 | 66 ± 7 | 70 ± 8 | 70 ± 8 | 73 ± 9 |
| Mean difference (95%CI) | -2 (-9, 5) | -1 (-6, 5) | -1 (-5, 3) | 1 (-3, 5) | 1 (-4, 6) | 1 (-3, 5) | 1 (-3, 5) |
| *P* value | 0.557 | 0.840 | 0.715 | 0.600 | 0.660 | 0.712 | 0.614 |
| Group | *F*= 0.004, *P*= 0.950 | | | | | | |
| Time | *F*= 26.279, *P*< 0.001 | | | | | | |
| Interaction | *F*= 0.384, *P*= 0.760 | | | | | | |

Values are presented as mean ± SD. A two-sample t-test for between-group difference comparisons was performed. Repeated measures analyses of variance for within-group comparisons were performed and post-hoc comparisons were made using the Bonferroni correction. T0, baseline; T1, 1 min after intubation; T2; after incision; T3, 1 min before osteotomy of the maxilla; T4, the maximum value measured during maxillary down-fracture; T5, 1min before osteotomy of the mandible; T6, the maximum value measured during osteotomy of the mandible. **P* < 0.05 compared to the T2; ^┼^ *P* < 0.05 compared to the T4.

**Table S6** The changes in mean arterial pressure in both groups at 7 measurement points

| Group | T0 | T1 | T2 | T3 | T4 | T5 | T6 |
| --- | --- | --- | --- | --- | --- | --- | --- |
| low volume group | 84 ± 8 | 76 ± 12 | 70 ± 10 | 68 ± 8 | 74 ± 9 | 66 ± 7^┼^ | 69 ± 8 |
| high volume group | 83 ± 7 | 77 ± 12 | 70 ± 10 | 66 ± 5 | 70 ± 8 | 67 ± 6 | 72 ± 7 |
| Mean difference (95%CI) | 1(-3, 4) | -1 (-7, 5) | -1 (-6, 4) | 2 (-1, 5) | 4 (0, 8) | -1(-5, 2) | -4 (-7, 0) |
| *P* value | 0.769 | 0.680 | 0.788 | 0.249 | 0.059 | 0.459 | 0.064 |
| Group | *F*= 0.001, *P*= 0.977 | | | | | | |
| Time | *F*= 39.429, *P*< 0.001 | | | | | | |
| Interaction | *F*= 1.670, *P*= 0.151 | | | | | | |

Values are presented as mean ± SD. A two-sample t-test for between-group difference comparisons was performed. Repeated measures analyses of variance for within-group comparisons were performed and post-hoc comparisons were made using the Bonferroni correction. T0, baseline; T1, 1 min after intubation; T2; after incision; T3, 1 min before osteotomy of the maxilla; T4, the maximum value measured during maxillary down-fracture; T5, 1min before osteotomy of the mandible; T6, the maximum value measured during osteotomy of the mandible. **P* < 0.05 compared to the T2; ^┼^ *P* < 0.05 compared to the T4.

**Table S7** The changes in systolic blood pressure in both groups at 7 measurement points

| Group | T0 | T1 | T2 | T3 | T4 | T5 | T6 |
| --- | --- | --- | --- | --- | --- | --- | --- |
| low volume group | 113 ± 10 | 105 ± 16 | 97 ± 13 | 95 ± 9 | 102 ± 11 | 94 ± 7^┼^ | 101 ± 9 |
| high volume group | 115 ± 10 | 106 ± 14 | 96 ± 11 | 91 ± 8* | 99 ± 9 | 95 ± 7 | 102 ± 8 |
| Mean difference (95%CI) | -2 (-7, 3) | 0 (-8, 6) | 0 (-6, 6) | 4 (0, 8) | 2(-3, 7) | -2 (-5, 2) | -1 (-5, 3) |
| *P* value | 0.357 | 0.809 | 0.902 | 0.063 | 0.340 | 0.327 | 0.649 |
| Group | *F*= 0.005, *P*= 0.946 | | | | | | |
| Time | *F*= 48.678, *P*< 0.001 | | | | | | |
| Interaction | *F*= 1.168, *P*= 0.326 | | | | | | |

Values are presented as mean ± SD. A two-sample t-test for between-group difference comparisons was performed. Repeated measures analyses of variance for within-group comparisons were performed and post-hoc comparisons were made using the Bonferroni correction. T0, baseline; T1, 1 min after intubation; T2; after incision; T3, 1 min before osteotomy of the maxilla; T4, the maximum value measured during maxillary down-fracture; T5, 1min before osteotomy of the mandible; T6, the maximum value measured during osteotomy of the mandible. **P* < 0.05 compared to the T2; ^┼^ *P* < 0.05 compared to the T4.

**Table S8** The changes in diastolic blood pressure in both groups at 7 measurement points

| Group | T0 | T1 | T2 | T3 | T4 | T5 | T6 |
| --- | --- | --- | --- | --- | --- | --- | --- |
| low volume group | 69 ± 9 | 63 ± 11 | 57 ± 8 | 56 ± 7 | 60 ± 11 | 53 ± 11^┼^ | 54 ± 12 |
| high volume group | 70 ± 7 | 63 ± 12 | 57 ± 10 | 52 ± 5 | 56 ± 7 | 54 ± 10 | 57 ± 8 |
| Mean difference (95%CI) | -1 (-5, 3) | 0(-6, 6) | 0(-5, 5) | 4 (1, 7) | 4(0, 9) | -1 (-7, 4) | -3 (-8, 2) |
| *P* value | 0.581 | 0.991 | 0.958 | 0.008 | 0.070 | 0.622 | 0.256 |
| Group | *F*= 0.137, *P*= 0.713 | | | | | | |
| Time | *F*= 27.230, *P*< 0.001 | | | | | | |
| Interaction | *F*= 1.706, *P*= 0.139 | | | | | | |

Values are presented as mean ± SD. A two-sample t-test for between-group difference comparisons was performed. Repeated measures analyses of variance for within-group comparisons were performed and post-hoc comparisons were made using the Bonferroni correction. T0, baseline; T1, 1 min after intubation; T2; after incision; T3, 1 min before osteotomy of the maxilla; T4, the maximum value measured during maxillary down-fracture; T5, 1min before osteotomy of the mandible; T6, the maximum value measured during osteotomy of the mandible. **P* < 0.05 compared to the T2; ^┼^ *P* < 0.05 compared to the T4.
